# On the Inside

**DOI:** 10.1093/plphys/kiab465

**Published:** 2021-12-04

**Authors:** Peter V Minorsky

**Affiliations:** School of Health and Natural Sciences, Mercy College, Dobbs Ferry, NY, USA

## Pectins in guard cell walls modulate stomatal function

The reversible expansion and contraction of guard cells, which cause changes in stomatal aperture, are dynamic processes that involve a balance between intracellular turgor pressure and the physical resistance of the guard cell wall. Adequate guard cell wall elasticity and strength are needed to ensure the functional demands of stomatal dynamics. Previous studies have shown that the cell walls of guard cells are rich in pectins and that these complex carbohydrates have a profound effect on stomatal function. For example, pectin degradation, particularly in the middle lamella, facilitates pore formation between two sister guard cells during the last stomatal developmental step, and the polar stiffening of guard cells affected by pectins leads to more efficient stomatal response to altered turgor pressure. Building on these findings, Guo et al. (pp. 2820–2836) now characterize the role of two Arabidopsis (*Arabidopsis thaliana*) galacturonosyltransferases, GAUT10 and GAUT11, in plant growth, stomatal development, and stomatal dynamics. They report that the closely related genes, *GAUT10 and GAUT11*, are highly expressed in guard cells and are involved in pectin synthesis in guard cell walls. *GAUT10*/*GAUT11* double mutants exhibited reduced pectin synthesis and altered dynamics of homogalacturonan (HG) demethylesterification, leading to larger stomatal complexes with smaller pore areas, increased stomatal dynamics, and plants with enhanced drought tolerance. These results provide insight into the molecular mechanism of guard cell wall properties that are critical for stomatal dynamics and highlight the role of GAUT10 and GAUT11 in regulating stomatal dimensions and dynamics through modulation of pectin biosynthesis and distribution in guard cell walls.

## A mite species adapts to arabidopsis in just 25 generations

During co-evolution with their hosts, generalist herbivores have evolved an innate ability to feed on a broad range of host plants that display a wide array of resistance traits, whereas specialist herbivores use highly efficient adaptation mechanisms against a limited set of host defenses. Although one might assume that a broadly generalist strategy, which allows for more potential food sources, would always be preferred, this is not necessarily the case because the host plants are not just a food resource but may also be sites of insect mating and oviposition. As a result, many generalist phytophagous insect populations are a composite of genotypes or subpopulations with slightly different host preferences. A case in point is the two-spotted spider mite (*Tetranychus urticae*), a generalist herbivore that feeds on over 1,100 plant species from more than 100 families. Such a wide host range indicates that *T. urticae* can counteract a great diversity of plant resistance traits. However, individual *T. urticae* populations do not perform equally well on all potential host plants. Instead, *T. urticae* has an outstanding ability to adapt to new hosts. The mechanism of this host adaptability is not known. Salehipourshirazi et al. (pp. 2608–2622) now examine *T. urticae* host adaptation following the shift of a bean (*Phaseolus vulgaris*)-adapted mite population to *Arabidopsis*. It has been hypothesized that expanded and functionally versatile detoxification capabilities enable generalist herbivores to cope with diverse allelochemicals and to feed on many host plants. In support of this idea, the authors show that in ∼25 generations of mite selection on Arabidopsis plants, mites evolved highly efficient detoxification-based adaptation involving changes in cytochrome P450 monooxygenases. Thus, specialization to plant resistance traits can occur within an ecological timescale.

## Melatonin functions in stomatal immunity

The production of Chinese ginseng (*Panax notoginseng*) is plagued by leaf diseases caused by *Alternaria panax and Pseudomonas syringae*. To prevent and control these diseases, a variety of fungicides and bactericides have been used during the production process, posing a potential risk to human health. One type of innate immunity involved in plants fighting against the invading pathogens is the recognition of pathogen- and microbe-associated molecular patterns (PAMP/MAMPs) through pattern recognition receptors (PRRs) on the cell surface, leading to basal resistance and a subsequent defense response known as PAMP/MAMPs-triggered immunity (PTI). PTI is associated with stomatal closure and reduced invasion by pathogens. Upon sensing MAMPs (e.g. bacterial flagellins), FLAGELLIN SENSING 2 (FLS2) interacts with its co-receptor BRASSINOSTEROID INSENSITIVE 1-associated kinase 1 (BAK1) that phosphorylates the heterotrimeric GTP-binding protein Gα subunit (GPA1), resulting in a reactive oxygen species (ROS) burst and stomatal immunity. Melatonin plays important role in plant disease response, but the mechanisms are largely unknown. Yang et al. (pp. 2837–2851) now show that melatonin functions in stomatal immunity in *P. notoginseng and Arabidopsis*. Biochemical analyses showed that melatonin-induced stomatal closure plays a prominent role in preventing invasion of *P.* *syringae* via activation of mitogen-activated protein kinase (MAPK) and NADPH oxidase-mediated ROS production. Putative phytomelatonin receptor 1 (PMTR1) is required for perceiving melatonin signaling in stomatal closure and activation of MAPK. Biochemical and genetic tests found PMTR1 is essential for flg22- and melatonin-induced MAPK activation. The application of melatonin induces stomatal closure. and reduces the bacterial growth in *fls2 and bak1* plants, indicating that PMTR1 acts as a downstream signaling component in FLS2- and BAK1-mediated stomatal immunity.

## Myosin’s role in polarized growth in moss

In tip-growing plant cells, growth results from the myosin XI- and F-actin-mediated deposition of cell wall polysaccharides transported by secretory vesicles. Previous studies have shown that myosin XI anticipates F-actin accumulation at the cell’s tip, suggesting a mechanism where vesicle clustering via myosin XI increases F-actin polymerization. Galotto et al. (pp. 2509–2529) have hypothesized that myosin XI causes the clustering of vesicles, thereby enabling local F-actin polymerization, and that this mechanism is essential for the maintenance of apical growth. To evaluate this model, the authors generated moss (*Physcomitrium patens*) plants harboring a myosin XI temperature-sensitive allele. They report that the loss of myosin XI function alters tip cell morphology, vacuolar homeostasis, and cell viability but only if F-actin is polymerized. Loss-of-function analysis shows that myosin XI focuses and directs vesicles at the tip of the cell, which induces formin-dependent F-actin polymerization, thereby increasing F-actin’s local concentration. These findings support the role of myosin XI in vesicle focusing, possibly via clustering and F-actin organization. The authors also conclude that myosin XI is involved in the maintenance of vacuole homeostasis and cell viability.

## Remote sensing identifies a senescence quantitative trait locus

Environmental stresses from climate change can alter source–sink relations during plant maturation, leading to premature senescence and decreased yields. Elucidating the genetic control of natural variations in senescence in wheat (*Triticum aestivum*) is facilitated by the use of unmanned aerial vehicle (UAV) based imaging techniques. Physiological attributes such as canopy chlorophyll content and photosynthesis and the impact of nutrients, water, and heat stress on plant growth can only be quantified non-destructively by measuring variations in the spectral reflectance. Thus, adapting field-based advanced remote sensing technology to phenotype physiological and biochemical traits beyond conventional traits that are assessed by eye is the future of crop breeding. Hassan et al. (pp. 2623–2636) now describe the use of UAVs to quantify senescence in wheat using vegetative indices derived from multispectral images. By means of UAV technology, the authors were able to detect senescence at various growth time points after anthesis in the field. Selecting for slow senescence using a combination of different UAV-based vegetation indices was found to be more effective than using a single ground-based vegetation index. The authors identified 28 quantitative trait loci (QTLs) for vegetative growth, senescence, and grain yield using a single-nucleotide polymorphism (SNP) array. Seventeen of these new QTLs for vegetation indices from UAV-based multispectral imaging were mapped. This integrated approach allowed for the identification of an important, previously unreported, senescence-related locus on chromosome 5D that showed high phenotypic variation for all UAV-based vegetation indices at all time points during grain filling. These results suggest that UAV-based high-throughput phenotyping is advantageous for the temporal assessment of the genetics underlying senescence in wheat.

## Phenotyping stomatal closure by thermal imaging

The synchrony of stomatal opening and closing with fluctuations in photosynthetic CO_2_ assimilation varies within and among species, with significant consequences for intrinsic water-use efficiency (iWUE, the ratio of net photosynthetic carbon assimilation (A) to the conductance of stomata to water vapor [*g_s_*]). If conditions are favorable for A, but *g_s_* is low, A will be limited by CO_2_ supply. Conversely, if A is low, but *g_s_* is high, unnecessary transpiration will occur with no corresponding benefit to A, substantially decreasing iWUE. Generally, *g_s_* responds an order of magnitude more slowly than A to a decrease in photosynthetic Photon Flux Density, declining to a new steady-state over the course of several minutes. This results in a de-synchronization of A and *g_s_* and a decrease in iWUE. It has been estimated that the synchronization of A and *g*s in their responses to fluctuating light, could yield substantial benefits to iWUE of 20%–30%. Accelerating stomatal movement by breeding or biotechnology for improved iWUE, however, remains an open challenge in cereal crops, including the C_4_ species, *Sorghum bicolor*. Pignon et al. (pp. 2544–2562) have used high-throughput thermal imaging to assess 10 traits describing stomatal conductance (*g_s_*) before, during, and after a stepwise decrease in light intensity for 659 sorghum accessions. *g_s_* traits varied substantially across the population and were moderately heritable. An integrated genome-wide and transcriptome-wide association study identified candidate genes putatively driving variation in stomatal conductance traits. Of the 239 unique candidate genes identified, 77 were orthologs of *Arabidopsis* genes related to functions implicated in WUE, including stomatal opening/closing (24 genes), stomatal/epidermal cell development (35 genes), leaf/vasculature development (12 genes), or chlorophyll metabolism/photosynthesis (8 genes). These findings suggest many avenues for further investigations of the genetic basis of WUE.

